# Network Meta-Analysis of Once Weekly Selinexor-Bortezomib-Dexamethasone in Previously Treated Multiple Myeloma

**DOI:** 10.36469/001c.27080

**Published:** 2021-08-25

**Authors:** Michael Dolph, Gabriel Tremblay, Adrienne M Gilligan, Hoyee Leong

**Affiliations:** 1 Purple Squirrel Economics, New York, NY, USA; 2 Karyopharm Therapeutics, Newton, MA, USA

**Keywords:** selinexor, xpovio, network meta-analysis, previously treated multiple myeloma, boretexomib, dexamethasone, relapsed multiple myeloma, multiple myeloma

## Abstract

**Background:** Despite the availability of new treatments, multiple myeloma (MM) is an incurable cancer with nearly all patients relapsing and undergoing multiple lines of treatment. Performing head-to-head comparisons of all treatment options is not feasible. Thus, network meta-analyses play an important role in allowing health-care decision makers to compare the effectiveness of treatment options.

**Objectives:** A Bayesian network meta-analysis (NMA) was developed from studies identified from a systematic literature review (SLR) to evaluate the efficacy of once weekly oral selinexor with once weekly bortezomib and low-dose dexamethasone (XVd) relative to other therapies in previously treated MM.

**Methods**: Ovid was systematically searched for phase 2-3 randomized clinical trials (RCTs) in MM that assessed progression-free survival (PFS), overall survival (OS) and overall response rates (ORR). Two population subsets were assessed: second-line patients (2L) and third-line or greater patients (3L+). Base case results compared all regimens against twice weekly bortezomib and dexamethasone (Vd) as the anchored comparator regimen.

**Results:** Forty-seven RCTs met inclusion. For 2L PFS, OS and ORR, XVd had, on average, out of all iterations, the 6th (out of 21), 4th (out of 15), and 5th (out of 20) best result, respectively, versus Vd. For 3L+ PFS, OS and ORR, XVd had the 12th (out of 24), 11th (out of 22), and 8th (out of 25) best result, respectively, versus Vd. There was no statistically significant difference between XVd and other top-ranking therapies for PFS, OS, and ORR in either 2L and 3L+ except for daratumumab/bortezomib/dexamethasone [DVd], which was favorable versus XVd (2L PFS only).

**Discussion:** Results for XVd were more favorable in 2L, having a higher probability of being a top 5 regimen, compared with 3L+ therapies based on the reported clinical trial results. However, in typical clinical practice, most triplet regimens have been modified using weekly bortezomib dosing, raising questions about the actual efficacy of these regimens versus the reported results using twice weekly bortezomib dosing.

**Conclusions:** The addition of XVd, which was designed with once weekly bortezomib dosing, to the treatment landscape for previously treated MM provides a regimen that may potentially be noninferior to the other top 5 regimens in both 2L and 3L+ settings and is associated with less peripheral neuropathy.

## BACKGROUND

Accounting for approximately 32 270 new cancer cases each year in the United States, multiple myeloma (MM) is the second most common hematologic malignancy after Non-Hodgkin’s lymphoma.^1–3^ MM is characterized by the abnormal growth of clonal plasma cells and pathogenic antibody production, resulting in renal impairment, anemia, bone fractures, hypercalcemia and susceptibility to infections.^4^ In the United States, MM is typically diagnosed at a median age of 69 years with five-year survival rates ranging from 71.7% in patients under 50 years of age to 32.9% in patients over 75 years of age.^2^

Despite the availability of new treatments, MM is an incurable cancer with nearly all patients relapsing and undergoing multiple lines of treatment, with each line providing diminishing effectiveness.^1^ Thus, the course of MM is typified by a series of increasingly short remissions and treatment-refractory relapses until death occurs. Given these recurring relapses, patients experience declining health-related quality of life (HRQoL) as treatment efficacy and duration of response weaken across subsequent lines of treatment.^1,5–8^ Additionally, MM continuously evolves through clonal evolution over the course of therapy, which requires drugs with novel mechanisms of action to overcome resistance.^9^

Initial therapy for MM typically includes a proteasome inhibitor (PI) and an immunomodulatory drug (IMiD), with or without an anti-CD38 monoclonal antibody (mAb); fit patients may then undergo high dose chemotherapy with stem cell transplantation. When disease relapses, the majority are treated again with a PI, an IMiD ±, and an anti-CD38 mAb with the primary goal to achieve disease control with acceptable toxicity and decent patient-defined quality of life.^10^ Essentially all patients develop MM refractory to these three drug classes within 1-3 lines of therapy. Several years ago, approaches to the treatment of triple-class refractory disease were limited and included conventional chemotherapy, salvage autologous stem cell transplantation, and recycling previous regimens, each of which have generally had short-lived efficacy.^11^ To optimize outcomes in MM, providers must consider patient age, comorbidities, cytogenetic risk, and response to prior therapies.^12^ Ultimately, however, drugs with novel mechanisms of action that are additive or synergistic with existing agents are required in order to effectively prolong duration and quality of life.

Selinexor (XPOVIO; Karyopharm Therapeutics, Inc.) is an oral, first-in-class selective inhibitor of nuclear export (SINE) that blocks Exportin 1 and inhibits the nuclear export of tumor suppressor proteins, growth regulators, and messenger ribonucleic acids (mRNAs) of oncogenic proteins.^13^ Nuclear retention of major tumor suppressor proteins and cell cycle regulators leads to their functional activation mediating oncogenic growth arrest and cancer cell apoptosis.^14^ Selinexor was initially approved by the US Food and Drug Administration (FDA) in combination with dexamethasone for the treatment of adult patients with relapsed or refractory MM who have received at least four prior therapies and whose disease is refractory to at least 2 PIs, at least 2 IMiDs, and an anti-CD38 mAb–penta-refractory MM. More recently, selinexor has been approved by the FDA in combination with bortezomib and dexamethasone for the treatment of adult patients with MM who have received at least 1 prior therapy based on results of the BOSTON trial.^13^ BOSTON was a Phase 3, open-label, randomized double-arm trial that compared the efficacy, safety, and HRQoL of once weekly oral selinexor with once weekly bortezomib and low-dose dexamethasone (XVd) versus standard twice weekly bortezomib plus moderate-dose dexamethasone (Vd) in adult patients with previously treated MM who have received 1 to 3 prior anti-MM regimens. Patients were randomized in a 1:1 allocation to 1 of 2 treatment arms (XVd or Vd). The once weekly bortezomib dosing was chosen based on the high level of synergy between the agents.^15^ The XVd regimen conferred a significant 47% increase in median progression-free survival (PFS), 49% increase in time to next therapy (TTNT) and higher overall response rates compared to Vd, with a trend to improved overall survival (OS, hazard ratio [HR] 0.84, *P*=0.1852). This is the first trial of a novel once-weekly bortezomib-based triplet therapy that showed lower rates of overall and Grade ≥ 2 peripheral neuropathy (PN), while conferring a longer PFS with ~35% fewer clinic visits, versus standard twice weekly Vd.^13^

Performing head-to-head comparisons of all treatment options is not feasible. Thus, network meta-analyses play an important role in allowing health-care decision makers to compare the effectiveness of treatment options.^16–18^ This manuscript reports the results of an NMA that compared the effectiveness of XVd to other second-line (2L) and third-line or greater (3L+) treatment options using outcomes most relevant to patients with previously treated MM: PFS, OS and overall response rates (ORRs).

## METHODS

A comprehensive systematic literature review (SLR) was performed to identify all randomized controlled trials (RCTs) of therapies for previously treated MM following the principles outlined in the Cochrane Handbook for Systematic Reviews of Interventions, the Guidance for Undertaking Reviews in Healthcare, and the National Institute for Health and Care Excellence (NICE) Decision Support Unit.^19,20^ The key biomedical literature databases (Medical Literature Analysis and Retrieval System Online [MEDLINE®], Excerpta Medica Database [Embase®]) and Cochrane collaboration were consulted. The inclusion and exclusion criteria are presented in Table 1.

**Table 1.** Inclusion and Exclusion Criteria

	**Inclusion**	**Exclusion**
**Patient Population**	• Patients diagnosed with relapsed or refractory multiple myeloma (RR MM)	• Non-human
		• Patients with newly diagnosed MM
		• Patients with other cancer types
**Intervention and Comparators**	• bortezomib	• Studies not including at least one of the intervention listed in the Inclusion Criteria
	• lenalidomide	
	• pomalidomide	
	• panobinostat	
	• ixazomib	
	• elotuzumab	
	• daratumumab	
	• carfilzomib	
	• selinexor	
**Outcome Measures**	• Overall survival (OS)	• Studies not including at least one of the outcomes listed in the Inclusion Criteria
	• Progression-free survival (PFS)	
	• Objective response rates (ORR)	
	• Safety	
	• Health related quality of life (HRQoL)	
	• Randomized clinical trials	• Non-human/pre-clinical studies
	• Sub-group analyses of previously published studies	• Reviews/Editorials/Notes/Comments/Letters
**Study Design**	• Systematic reviews and meta-analyses (for cross-checking only)	• Non-interventional studies
		• Retrospective studies
	• Pooled Analyses (for cross-checking only)	• Observational studies
		• Uncontrolled studies
		• Phase 1 Dose escalation study or pharmacokinetics study
		• Case series
		• Case reports
	• English language	• Non-English language studies
	• Year limitation: 2016-current	
		• Case reports

For these analyses, Bayesian NMA models were used since the comparison networks contained closed links to multiple different treatments (ie, the analyses involved a mixed treatment comparison). For the results of NMA to be valid, transitivity (potential modifiers of treatment effects are similarly distributed across trials) and consistency (indirect effect estimates are consistent with those of direct effects) should be present. Therefore, careful evaluation of clinical and methodological heterogeneity across trials was assessed to ensure that network transitivity was maintained (ie, similar patients and characteristics within and across trials).

To perform the NMAs, R statistical software was used with PFS HRs, OS HRs and the proportion of patients achieving ORR from high-quality relatively homogenous RCTs. Models used non-informative priors and were initiated after “burning-in” 40 000 iterations while posterior distributions were based on 200 000 iterations. Results were presented as point estimates (in the form of NMA-adjusted HRs, ORs or risk ratios [RRs]) with 2-sided credible intervals of 95%, and as favorability-ranked outputs. The data and the results were verified by 2 different quality control agents. All methods were in line with the Cochrane Review Guidelines.

Treatment network formation for each therapy line and outcome were made wherein 6 total networks were developed: 2L PFS, 2L OS, 2L ORR, 3L+ PFS, 3L+ OS, and 3L+ ORR. In these networks, PFS and OS outcomes were assessed as HRs, and ORR were assessed as RRs. An ad hoc scenario analysis was also performed that was limited to studies with regimens containing bortezomib treatment backbones only. This analysis is particularly relevant as most patients are treated with a lenalidomide-based frontline regimen, and therefore 2L and 3L+ therapies for these patients rarely contain lenalidomide.

## RESULTS

**Figure 1.**
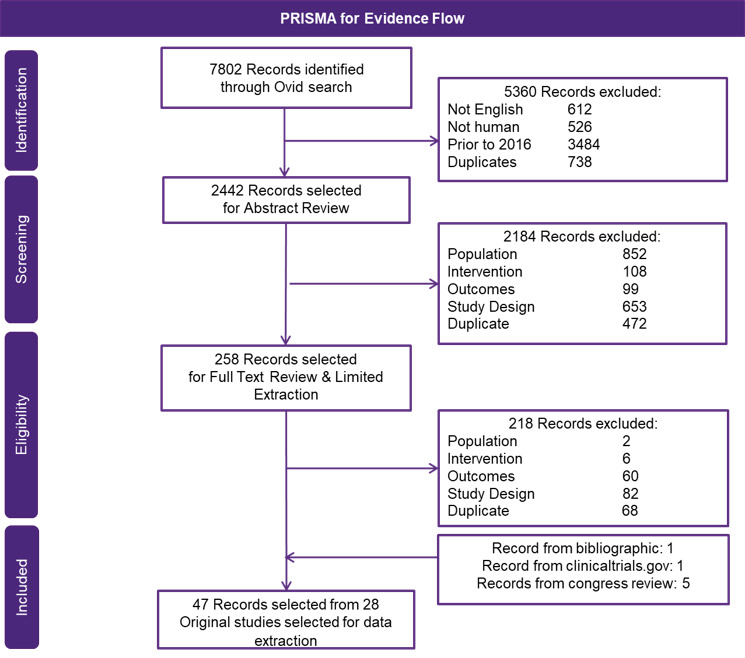
PRISMA Diagram

**Figure 2.**
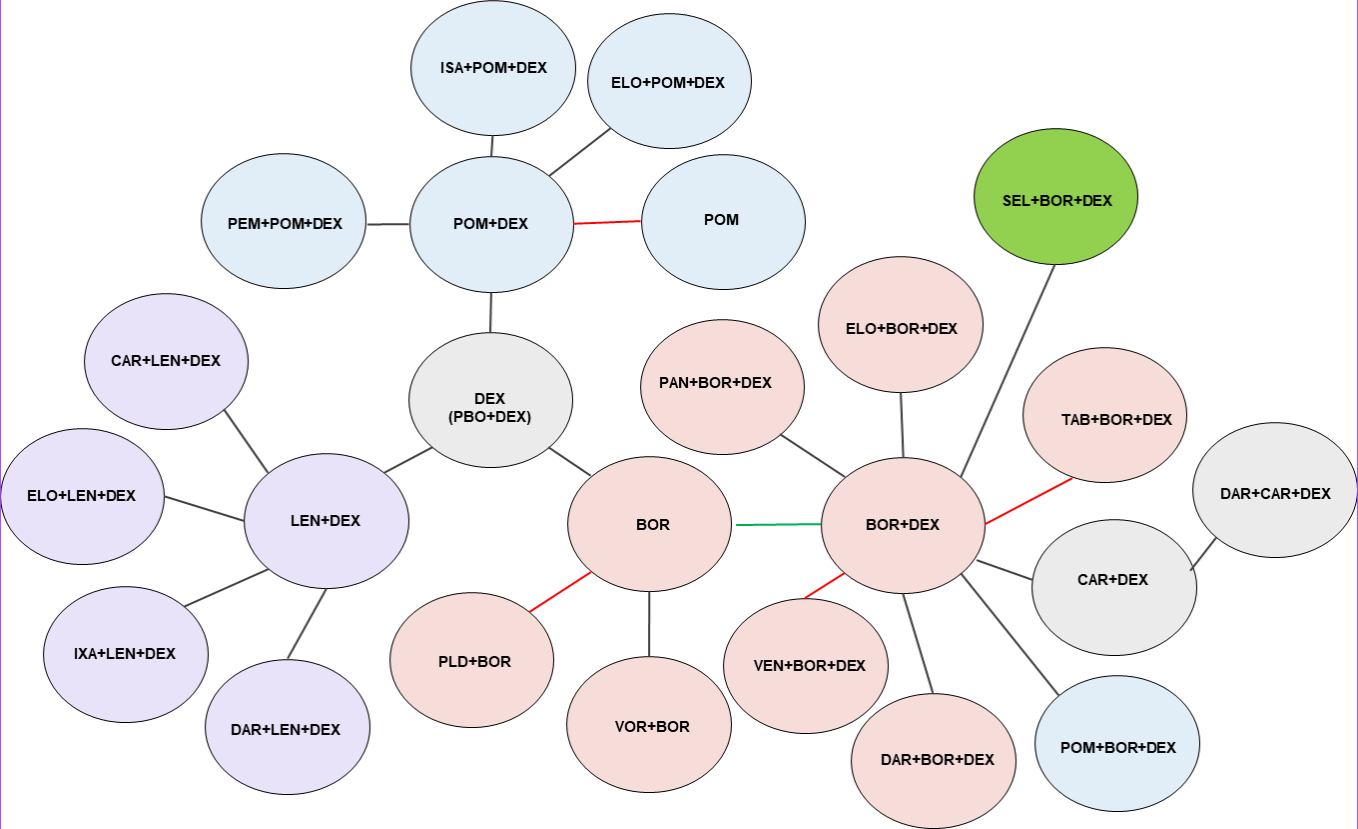
Comparison Network (3L+ network for PFS) Note: Figure 2 represents the 3L+ PFS NMA network. Networks varied between treatment line and outcome assessed. Black line: Normal RCT connection with pure 3L+ population. Red line: RCT connection with mixed 2L/3L+ population, but majority 3L+. Green line: Retrospective study. Abbreviations: 2L, second-line patients; 3L+, third-line or greater patients; BOR, bortezomib; AR, carfilzomib; DAR, daratumuab; DEX, dexamethasone; ELO, elotuzumab; ISA, isatuximab; IXA, ixazomib; LEN, lenalidomide; NMA, network meta-analyses; PAN, panobinostat; PEM, pembrolizumab; PFS, progression-free survival; PLD, pegylated liposomal doxorubicin; POM, pomalidomide; RCT, randomized controlled trial; SEL, selinexor; TAB, tabalumab; VOR, vorinostat.

From the SLR, a total of 7802 records were identified, and of those, 47 records were selected (Table S1 and Table S2), where a total of 28 phase 2 or 3 RCTs met the inclusion criteria and were extracted (Figure 1). Assessment and stratification of studies were based on treatment line (ie, 2L and 3L+). Studies that included patients in multiple treatment lines were stratified based on the category holding the majority of patients (ie, a study containing >50% of 3L+ patients would be included in the 3L+ network) (Figure 2). For all 6 networks, 1 retrospective match-paired analysis (Dimpopoulos, 2015) was required to connect bortezomib (BOR) with Vd (BOR+DEX).^21^ As such, the additional scenario analysis only including regimens with a Vd backbone was performed, which did not rely on the inclusion of this retrospective study. For the 3L+ networks, 2 comparators (cyclophosphamide/dexamethasone [CYC+DEX] and carfilzomib [CAR]) were excluded, as they had no connection to the main network.

**Figure 3.**
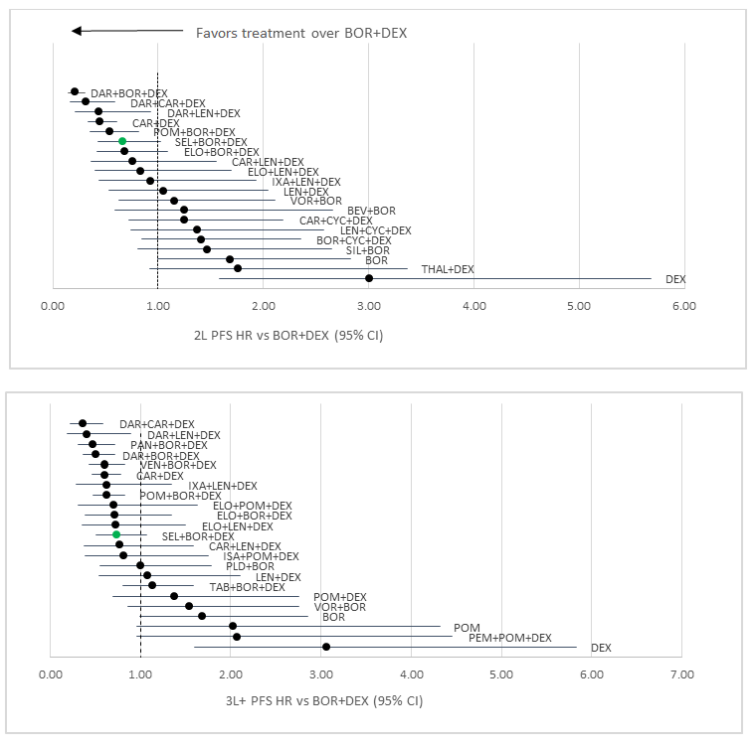
PFS Results (stratified by treatment line) Abbreviations: BOR+DEX, bortezomib + dexamethasone; DAR+BOR+DEX, daratumumab + bortezomib + dexamethasone; DAR+CAR+DEX, daratu- mumab + carfizomib + dexamethasone; DAR+LEN+DEX, daratumumab + lenalidomide + dexamethasone; CAR+DEX, carfizomib + dexamethasone; POM+BOR+DEX, pomalidomide + bortezomib + dexamethasone; SEL+BOR+DEX, selinexor + bortezomib + dexamethasone; ELO+BOR+DEX, elotuzum- ab + bortezomib + dexamethasone; CAR+LEN+DEX, carfizomib + lenalidomide + dexamethasone; ELO+LEN+DEX, elotuzumb + lenalidomide + dexa- methasone; IXA+LEN+DEX, ixazomib + lenalidomide + dexamethasone; LEN+DEX, lenalidomide + dexamethasone; VOR+BOR, vorinostat + bortezomib; BEV+BOR, bevacizumab + bortezomib; CAR+CYC+DEX, carfilzomib + cyclophosphamide + dexamethasone; LEN+CYC+DEX, lenalidomide + cyclophos- phamide + dexamethasone; BOR+CYC+DEX, bortezomib + cyclophosphamide + dexamethasone; SIL+BOR, siltuzimab + bortezomib; BOR, bortezomib; THAL+DEX, thalidomide + dexamethasone; DEX, dexamethasone; PAN+BOR+DEX, panobinostat + bortezomib + dexamethasone; VEN+BOR+DEX, venetoclax + bortezomib + dexamethasone; ELO+POM+DEX, elotuzumab + pomalidomide + dexamethasone; ISA+POM+DEX, isatuximab + pomalido- mide + dexamethasone; PLD+BOR, pegylated liposomal doxorubicin + bortezomib; TAB+BOR+DEX, tabalumab + bortezomib + dexamethasone; POM+- DEX, pomalidomide + dexamethasone; POM, pomalidomide; PEM+POM+DEX, pembrolizumab + pomalidomide + dexamethasone.

For the 2L PFS network, 21 studies were included. The majority of included studies (14) were RCTs with exclusively 2L outcomes reported. Five included studies were a mixed population with the majority 2L patients but some 3L+ patients, while 2 were primarily 3L+ patients (required to connect dexamethasone [DEX] with lenalidomide/dexamethasone [LEN+DEX]). XVd had the 6th best HR result versus Vd out of 21 regimens assessed for 2L PFS (HR: 0.66; 95% CI: 0.43-1.02). Out of the five therapies that were favorable to XVd, only 1 (daratumumab/bortezomib/dexamethasone [DAR+BOR+DEX], DVd) had a statistically significant improvement versus XVd (Figure 3). Importantly, the activity of the DVd regimen is based on twice weekly bortezomib dosing in the CASTOR study, which is rarely incorporated in current clinical practice. Moreover, the CASTOR study required that bortezomib was discontinued after 24 weeks in both the DVd and Vd arms, again not consistent with usual clinical practice and magnifies the effects of daratumumab after the first 24 weeks where no therapy was given on the control arm. In 2L PFS, XVd had an average rank of 7.2 out of 21 regimens included, with the highest probability of being ranked 6th overall and a 24.5% probability that XVd was a top 5 regimen (Table S3).

**Figure 4.**
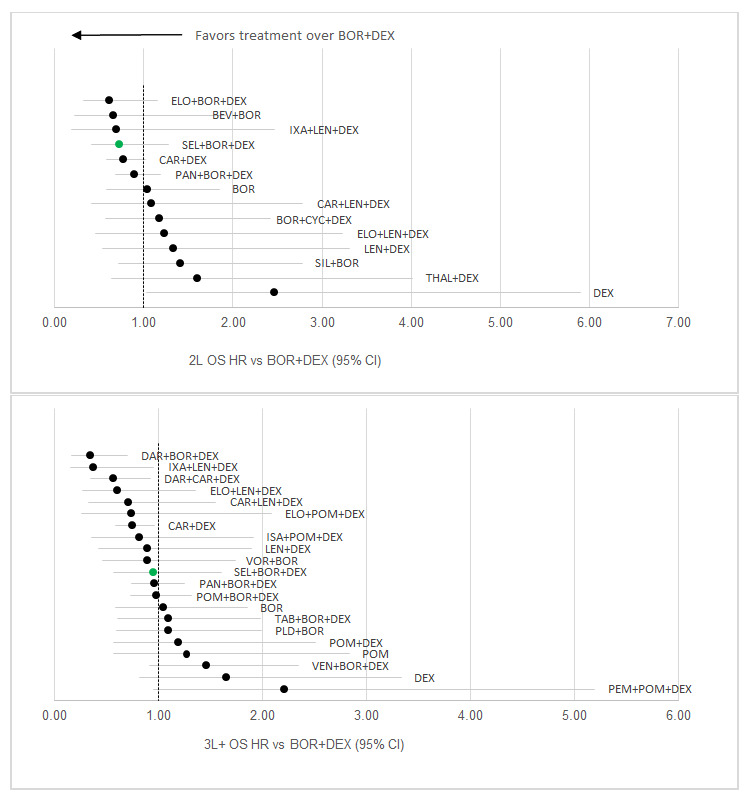
OS Results (stratified by treatment line) Abbreviations: BOR+DEX, bortezomib + dexamethasone; ELO+BOR+DEX, elotuzumab + bortezomib + dexamethasone; BEV+BOR, bevacizumab + bortezomib; IXA+LEN+DEX, ixazomib + lenalidomide + dexamethasone; SEL+BOR+DEX, selinexor + bortezomib + dexamethasone; CAR+DEX, carfi- zomib + dexamethasone; PAN+BOR+DEX, panobinostat + bortezomib + dexamethasone; BOR, bortezomib; CAR+LEN+DEX, carfizomib + lenalidomide + dexamethasone; BOR+CYC+DEX, bortezomib + cyclophosphamide + dexamethasone; ELO+LEN+DEX, elotuzumb + lenalidomide + dexamethasone; LEN+DEX, lenalidomide + dexamethasone; SIL+BOR, siltuzimab + bortezomib; THAL+DEX, thalidomide + dexamethasone; DEX, dexamethasone; DAR+BOR+DEX, daratumumab + bortezomib + dexamethasone; DAR+CAR+DEX, daratumumab + carfizomib + dexamethasone; ELO+POM+DEX, elotuzumab + pomalidomide + dexamethasone; ISA+POM+DEX, isatuximab + pomalidomide + dexamethasone; VOR+BOR, vorinostat + bortezomib; POM+BOR+DEX, pomalidomide + bortezomib + dexamethasone; TAB+BOR+DEX, tabalumab + bortezomib + dexamethasone; PLD+BOR, pegylated liposomal doxorubicin + bortezomib; POM+DEX, pomalidomide + dexamethasone; POM, pomalidomide; VEN+BOR+DEX, venetoclax + bortezomib + dexamethasone.

For the 2L OS network, 15 studies were included but relatively few (4) exclusively reported 2L OS. Nine included studies were a mixed population with the majority 2L but some 3L+ patients, while 1 was primarily 3L+ patients (required to connect DEX with LEN+DEX). XVd had the 4th best HR result versus Vd out of 15 regimens assessed (HR: 0.73; 95% CI: 0.42- 1.28) (Figure 4). In 2L OS, XVd had an average rank of 4.6 out of 15 regimens included, with the highest probability of being ranked 2nd overall and an 11.4% probability of being ranked 1st overall. There was a 68.6% probability of XVd being a top 5 regimen (Table S4). Out of the three therapies that were more favorable than XVd, none had a statistically significant difference versus XVd.

**Figure 5.**
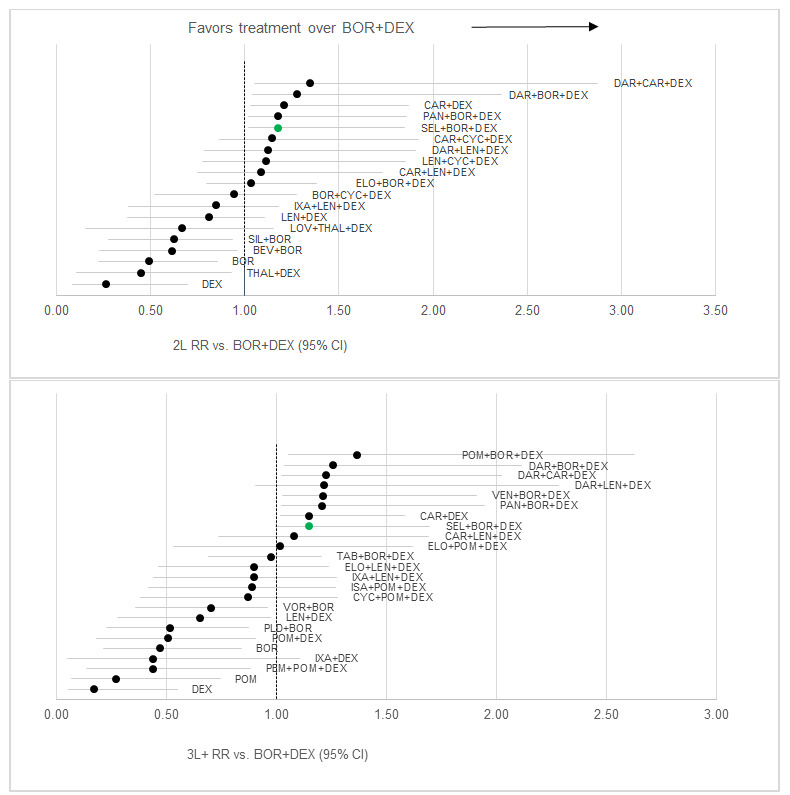
ORR Results (stratified by treatment line) Abbreviations: BOR+DEX, bortezomib + dexamethasone; DAR+CAR+DEX, daratumumab + carfizomib + dexamethasone; DAR+BOR+DEX, daratumum- ab + bortezomib + dexamethasone; CAR+DEX, carfizomib + dexamethasone; PAN+BOR+DEX, panobinostat + bortezomib + dexamethasone; SEL+BOR+- DEX, selinexor + bortezomib + dexamethasone; CAR+CYC+DEX, carfilzomib + cyclophosphamide + dexamethasone; DAR+LEN+DEX, daratumumab + lenalidomide + dexamethasone; LEN+CYC+DEX, lenalidomide + cyclophosphamide + dexamethasone; CAR+LEN+DEX, carfizomib + lenalidomide + dexamethasone; ELO+BOR+DEX, elotuzumab + bortezomib + dexamethasone; BOR+CYC+DEX, bortezomib + cyclophosphamide + dexamethasone; IXA+LEN+DEX, ixazomib + lenalidomide + dexamethasone; LEN+DEX, lenalidomide + dexamethasone; LOV+THAL+DEX, lovastatin + thalidomide + dexamethasone; SIL+BOR, siltuzimab + bortezomib; BEV+BOR, bevacizumab + bortezomib; BOR, bortezomib; DEX, dexamethasone; POM+BOR+DEX, pomalidomide + bortezomib + dexamethasone; VEN+BOR+DEX, venetoclax + bortezomib + dexamethasone; ELO+POM+DEX, elotuzumab + pomalid- omide + dexamethasone; TAB+BOR+DEX, tabalumab + bortezomib + dexamethasone; ELO+LEN+DEX, elotuzumb + lenalidomide + dexamethasone; ISA+POM+DEX, isatuximab + pomalidomide + dexamethasone; CYC+POM+DEX, cyclophosphamide + pomalidomide + dexamethasone; VOR+BOR, vorinostat + bortezomib; PLD+BOR, pegylated liposomal doxorubicin + bortezomib; POM+DEX, pomalidomide + dexamethasone; IXA+DEX, ixazomib + dexamethasone; PEM+POM+DEX, pembrolizumab + pomalidomide + dexamethasone; POM, pomalidomide.

For the 2L ORR network, 20 studies were included and the majority (12) were RCTs with exclusively 2L outcomes reported. Eight included studies were a mixed population with the majority 2L patients but some 3L+ patients. XVd had the 5th best result versus Vd out of 20 regimens assessed (RR: 1.18; 95% CI: 1.02-1.85) (Figure 5). Out of the four therapies that were favorable to XVd, none had a statistically significant improvement versus XVd. In 2L ORR, XVd had an average rank of 5.6 out of 20 regimens included, with the highest probability of being ranked 5th overall. There was a 50.1% probability of XVd being a top 5 regimen (Table S5).

Twenty-four studies were included in the 3L+ PFS network, with the majority (19) having exclusively 3L+ outcomes reported. Four included studies were a mixed population with the majority 3L+ patients but some 2L patients, and 1 was exclusively 2L patients required to link Vd with BOR. In 3L+ PFS, XVd had the 12th best HR result versus Vd out of 24 regimens assessed (HR: 0.74; 95% CI: 0.50-1.08) (Figure 3). XVd had an average rank of 10.7 out of 24 regimens included, with the highest probability of being ranked 9th overall. There was a 7.6% probability that XVd was a top 5 regimen (Table S6). Out of the 11 therapies that were favorable to XVd, none had a statistically significant difference versus XVd.

For the 3L+ OS network, of the 22 included studies, 11 reported 3L+ outcomes while a large number (10) were a mixed population with the majority 3L+ patients but some 2L patients. As with the 3L+ PFS network, 1 study exclusively reported 2L results, which was required to link BOR with Vd. In 3L+ OS, XVd had the 11th best HR result versus Vd out of 22 regimens assessed (HR: 0.95; 95% CI: 0.56-1.60) (Figure 4). XVd had an average rank of 12.0 out of 22 regimens included, with the highest probability of being ranked 9th overall. There was a 12.8% probability that XVd was a top 5 regimen (Table S7). Out of the 10 therapies that were favorable to XVd, none had a statistically significant difference versus XVd.

For the 3L+ ORR network, 27 studies were included. The majority of studies (17) had exclusively 3L+ outcomes reported. Nine included studies were a mixed population with the majority 3L+ patients but some 2L patients, and 1 study exclusively reported 2L results, which was required to link BOR with Vd. XVd had the 8th best result versus Vd out of 25 regimens assessed (RR: 1.15; 95% CI: 1.00-1.70) (Figure 5). In 3L+ ORR, XVd had an average rank of 7.1 out of 25 regimens included, with the highest probability of being ranked 7^th^ overall. There was a 27.8% probability that XVd was a top 5 regimen (Table S8). Out of the seven therapies that were favorable to XVd, none had a statistically significant improvement versus XVd.

An additional NMA was performed that only included regimens containing Vd. This additional analysis avoided the need for the retrospective study to link between BOR and Vd and also allowed for comparisons between regimens with 1 shared common anchor (ie, all included studies in this additional analysis directly compared to Vd). Moreover, this analysis is highly relevant to current clinical practice where lenalidomide is used in the vast majority of frontline regimens and is, therefore, not used in most 2L and 3L+ regimens, meaning that lenalidomide-based combinations are less relevant to current treatment of relapsed MM. For 2L PFS, OS and ORR, XVd had the 3^rd^ best (out of 6), 2^nd^ best (out of 5), and 3^rd^ best (out of 6) result, respectively, versus Vd. This translated to a 58.6%, 84.8%, and 50.1% probability that XVd would be a top 3 ranked regimen when assessing 2L PFS, OS and ORR, respectively. For 3L+ PFS, OS and ORR, XVd had the 6^th^ best (out of 8), 2^nd^ best (out of 7), and 5^th^ best result (out of 7), respectively, versus Vd. This translated to a 13.5%, 50.7%, and 20.8% probability that XVd would be a top 3 ranked regimen when assessing 3L+ PFS, OS and ORR, respectively.

## DISCUSSION

As essentially all patients with MM are expected to inevitably relapse, the treatment landscape for the disease has seen the development of a plethora of options. Since the number of treatment options and lack of multi-arm randomized studies makes head-to-head comparisons challenging, NMAs enable the pooling of direct and indirect evidence from multiple studies, allowing comparison of treatments that were not directly compared in head-to-head trials.^16–18^

Current clinical practice guidelines from the Mayo Clinic and the European Society for Medical Oncology both recommend that patients experiencing their first relapse after an IMiD-based (typically lenalidomide) induction therapy move to IMiD-free regimens such as bortezomib and dexamethasone in 2L.^22,23^ The combination of bortezomib, a first-in-class PI, and dexamethasone is standard therapy for patients with MM.^24^ However, its twice-weekly dosing regimen is associated with potentially irreversible high rates of PN and twice weekly clinic visits, limiting its prolonged use and imposing substantial patient burden.^24^ PN can be minimized with once-weekly bortezomib therapy, and many physicians employ once weekly bortezomib-based regimens in the treatment of relapsed MM.^25^ Thus, developing therapies with novel mechanisms of action that demonstrate clinical benefits with once weekly bortezomib will serve a current and rapidly growing unmet medical need in patients with previously treated MM.^26–28^

As an oral, first-in-class SINE compound, selinexor works in combination with bortezomib (and other PIs) and dexamethasone to synergistically and selectively kill malignant plasma cells.^13,29^ The once-weekly XVd regimen, in which weekly oral selinexor replaces 1 of the twice-weekly doses of bortezomib, represents a simplification of bortezomib-based triplets for previously treated MM. Moreover, the once weekly XVd regimen, which has been adopted in routine clinical practice, was directly studied against standard twice weekly Vd in the BOSTON trial and is one of the few randomized studies to directly compare a “real world” bortezomib-based regimen against the twice weekly Vd regulatory standard. Compared to Vd and other Vd-based triplets, XVd is associated with reduced neuropathy rates and requires fewer clinic visits.^25,28^ Finally, selinexor does not require antimicrobial or antithrombotic prophylaxis, nor is it known to have deleterious cardiac effects, making it a good partner with other anti-MM agents.

This NMA allowed the comparison of the current treatment options for 2L and 3L+ MM patients. Overall, results for XVd were more favorable when assessing the 2L treatment networks, compared with the 3L+ networks. XVd results were strong for 2L OS (4th best HR overall versus Vd, at 0.73, with a 69% probability of being a top 5 regimen) and ORR (5th best RR overall versus Vd, at 1.18, with a 50% probability of being a top 5 regimen). XVd had the 6th best PFS HR result versus Vd out of 21 regimens assessed (HR: 0.66; 95% CI: 0.43-1.02). Importantly, of the 5 therapies that were favorable to XVd, only 1, 2L DVd had a statistically significant improvement versus XVd (in PFS only). It should be noted that the once weekly use of Vd in the XVd arm in the BOSTON trial was associated with considerably less PN (32.0%) than twice-weekly Vd in the DVd arm in the CASTOR trial (47.0%).^27,30^ In addition, the CASTOR trial had 2 important differences compared to routine clinical practice: (i) bortezomib was dosed twice weekly in both treatment arms, while once weekly bortezomib dosing is employed in most routine clinical practice; and (ii) bortezomib was discontinued in both arms after 24 weeks, essentially comparing daratumumab with no treatment after this period; this is also not typical of routine clinical practice.^27^

The additional analysis, which included only regimens containing Vd backbone therapies, was performed to compare more similar treatment options. Similar results were seen in this analysis. Out of the 2 therapies that were favorable to XVd in 2L PFS, only 1 (DVd) had a statistically significant improvement versus XVd. For 2L and 3L+ OS and ORR, there were no treatment options that had a statistically significant improvement versus XVd.

A basic strategy in MM, which is employed for other complex diseases, is to utilize novel mechanisms whenever possible when disease progression occurs. Thus, patients whose MM relapses are often treated with other agents besides bortezomib, including the IMiD pomalidomide, the SLAM/F7 mAb elotuzumab, an anti-CD38 mAb (daratumumab or isatuximab) and/or the more potent PI carfilzomib. Although patients may be treated with bortezomib in the front-line setting, they are often not treated through disease progression, either because they undergo stem cell transplantation or develop significant toxicity, typically PN. Thus, patients eligible for transplantation will receive their stem cell transplant while their disease remains sensitive to bortezomib. Furthermore, patients who are transplant ineligible with MM sensitive to bortezomib are frequently treated with an IMiD combination therapy and then transitioned to lenalidomide maintenance. Therefore, in the 2L or 3L+ setting, XVd can be utilized as a “mechanism-switch” approach to limit recycling of therapies with the same mechanism of action. In addition, while there are some differences in the mechanisms of resistance between carfilzomib and bortezomib, results have shown clinical responses with bortezomib-based therapies in carfilzomib refractory MM.^31^ This allows for further benefit of XVd among patients whose MM is refractory to a PI. These findings suggest that XVd can emerge as a standard approach with a new mechanism of action in the 2L or 3L+ setting following daratumumab therapy, given that XVd was noninferior to other therapies other than DVd in 2L PFS in this setting. Again, a lack of overlacking toxicities with other anti-MM agents allows for flexibility and broad use of the selinexor-containing regimen.

While the 2L studies had a slightly longer mean follow-up time than the 3L+ studies (26.3 months versus 22.7 months, respectively), other baseline characteristics were generally similar between the 2 groups (mean age 64.2 years versus 64.6 years, 56.4% male versus 55.2% male), which could suggest that the resulting differences between 2L and 3L+ studies may be due to exposure to prior lines of therapy, given that the characteristics of previous therapy can have major influences on the treatment effect of later interventions.^32^ Differences in these studies may also be attributed to the proportion of elderly/frail populations and/or patients with high-risk cytogenetics. In the BOSTON trial, more than half of patients in the XVd arm (50.5%) and 48.5% in the Vd arm reported high-risk cytogenetics. Furthermore, OS among the elderly population was significantly longer in the XVd arm compared to Vd (HR: 0.63, 95% CI: 0.39-1.02; *P*=0.029) and approaching significance among frail patients (HR: 0.62, 95% CI: 0.34-1.14; *P*=0.061). The reduced effect of XVd on OS in younger and more fit patients is expected given the large number of alternative treatments and prolonged expected survival in this population. If the data are available, future analysis stratified by the type of prior therapy received could also help shed light on the reason for these between-line differences. Additionally, some of the included regimens did not have data for certain outcomes in both the 2L and 3L+ population, requiring them to be excluded from that analysis.

These findings suggest that XVd is non-inferior or similar to all compared treatment options in the 2L and 3L+ setting, with the exception of DVd in 2L PFS, in the context of the caveats of the CASTOR study that bortezomib was discontinued after 24 weeks in both (DVd and Vd) treatment arms, which is rarely incorporated in current clinical practice. Futhermore, approximately half of the patients in the BOSTON trial had high risk cytogenetics, a subpopulation higher than that in other studies and an inherently more difficult-to-treat population. This, combined with its more convenient treatment regimen (ie, once weekly bortezomib) and substantially reduced bortezomib-induced PN compared to other Vd backbone therapies, support the role of XVd in providing patients a valuable treatment option for previously treated MM. Given that selinexor can work in combination with all anti-MM drug classes including PIs, IMiDs, and mAbs, (eg, with carfilzomib and dexamethasone [XKd]; pomalidomide and dexamethasone [XPd]; or daratumumab and dexamethasone [XDd]), clinicians have the opportunity to employ a unique mechanism of action in combination with existing backbone therapies to treat patients refractory to their primary therapy.^13,15,29,33,34^

Future analyses including a larger sample of studies for each regimen, if they were to become available, would likely improve the precision of the estimates and could allow for adjustment of potentially important effect-modifiers. Furthermore, additional studies could help improve the network links between regimens by reducing the need for non-RCT trials or trials with non-transitive populations to be used.

Results from our study align with previous findings in NMAs with previously treated MM.^32,35–37^ Weisal et al. compared OS, PFS, and ORR across IMid-free regimens, an analysis similar to our sub-group analysis of Vd backbone therapies only. Their findings similarly showed little statistical differences between treatments compared, with the exception of DVd, which significantly prolonged PFS compared with other IMiD-free regimens (such as carfilzomib plus dexamethasone [Kd], panobinostat plus Vd [FVd], cyclophosphamide plus Vd [CVd], and Vd). FVd and Kd demonstrated a statistical advantage in improving PFS compared with Vd. In terms of OS, DVd showed statistically significant improvements compared with Vd, but other comparisons were inconclusive. DVd and Kd showed significant improvements in ORR compared to Vd.^32^A more recent NMA by Arcuri et al, reviewed phase 3 trials with control arms that included lenalidomide or bortezomib (considering them equivalent therapies) and similarly found the compared therapies (daratumumab, pegylated liposomal doxorubicin, isatuximab, carfilzomib, pomalidomide, panobinostat, venetoclax, ixazomib, XVd, elotuzumab, cyclophosphamide, vorinostat, high-dose chemotherapy, ld, and Vd) to be similar in PFS and OS, with the exception of daratumumab triplet regimens, which performed significantly better.^35^

Selinexor triplet combinations including XDd and XPd are recommended on the NCCN Guidelines for previously treated MM.^7^ XDd, a PI and IMiD-free regimen, is well-tolerated and shows efficacy in patients with previously treated MM who had at least 1 prior line of therapy including a PI and an IMiD but whose disease is naïve to daratumumab.^29^ Similarly, XPd is an all-oral PI-sparing regimen that shows efficacy in patients with both pomalidomide- and lenalidomide-refractory MM.^38^ These combinations offer greater opportunities for class-switching and can delay repeated use of PIs and/or IMiDs. The inclusion of these combinations in future NMAs would provide a clearer understanding of their relative efficacy and place in therapy. Current clinical evidence suggests that once weekly oral selinexor in these various combinations offers deep and durable responses with potentially greater convenience and significantly fewer clinical visits.^13,33^

### Limitations

While the presented analysis is based on robust methods and consists of data obtained from a thorough SLR, there were some key limitations to note. First, there was a limited number of studies, particularly for certain treatment regimens where only 1 study was available. Furthermore, some of the included studies had very small sample sizes, which added to the overall uncertainty of the NMA results. Additionally, for some of the included comparators, data were not available for all assessed endpoints (eg, both PFS and OS HRs), which required those comparators to be excluded from some of the performed NMAs. Secondly, the treatment networks were relatively complex where certain links to the base case regimen comprised of up to 5 distal links. Therefore, variability was likely inflated due to the complexity of the network. Additionally, and relating to this point, there was a missing link in the network that required a retrospective study (Dimopoulos, 2015) to be used to link bortezomib with Vd.^21^ Inherently, the inclusion of this study did not satisfy the transitivity assumption as all other studies were prospective RCTs. However, since the connection between bortezomib and Vd was a key link in the network to allow for comparison to many of the included regimens, the use of this retrospective study was deemed necessary. Many of these limitations exist and were noted in other NMAs of previously treated MM.^32,35–37^

## CONCLUSION

The addition of XVd to the treatment landscape for previously treated MM provides a novel, convenient regimen that appears to be noninferior to other top 5 regimens in both 2L and 3L+ settings, and allows for a fourth (after IMiD, PI, and anti-CD38 mAb) novel mechanism to be leveraged against recurrent MM. Moreover, XVd may require the least clinic time as compared to other triplet regimens that incorporate a parenteral agent. These findings, combined with its highly convenient treatment regimen, and reduced bortezomib-induced PN, support XVd’s role in providing patients a promising treatment option for previously treated MM.^15,33,34^

## Supplementary Material

Online Supplementary Materials
