# Supplementary material for: Network Meta-Analysis of Once Weekly Selinexor-Bortezomib-Dexamethasone in Previously Treated Multiple Myeloma
Source: J Health Econ Outcomes Res. 2021 Aug 25;8(2):26–35. doi: 10.36469/001c.27080 (PMC8460427; doi:10.36469/001c.27080)
Supplement: Online Supplementary Materials [file jheor_2021_8_2_27080_70932.pdf]

Journal of Health Economics and Outcomes Research

Supplementary Online Material

Dolph M, Tremblay G, Gilligan AM, Leong H. Network meta-analysis of once weekly selinexor-bortezomib-dexamethasone in previously treated multiple myeloma. *JHEOR*. 2021;8(2):26-35.
doi:10.36469/jheor.2021.27080

Table S1. Study Characteristics (2L Population)
Table S2. Study Characteristics (3L+ Population)
Table S3. 2L PFS Ranking Probability Results
Table S4. 2L OS Ranking Probability Results
Table S5. 2L ORR Ranking Probability Results
Table S6. 3L+ PFS Ranking Probability Results
Table S7. 3L+ OS Ranking Probability Results
Table S8. 3L+ ORR Ranking Probability Results

This supplementary material has been provided by the authors to give readers additional information about their work.

This is an open-access article distributed under the terms of the Creative Commons Attribution 4.0 International License (CCBY-4.0). View this license's legal deed at http://creativecommons.org/licenses/by/4.0 and legal code at http://creativecommons.org/licenses/by/4.0/legalcode for more information.

Table S1. Study Characteristics (2L Population)

Short Reference	Study Acronym	Study Design	Study Population	Interventions Acronym	Tx Dosage Included (mg)	Follow-Up (median months)	Duration of Treatment (median months)	N	Age (median)	Male (%)
Dimopoulos_ASCO_2020 (abstract)	BOSTON (NCT03110562)	P3, RCT, open-label, multicenter	previously treated MM with 1 to 3 prior LOT	SEL+BOR+DEX	SEL: 100 mg<br>BOR: 1.3 mg/m^2<br>DEX: 20 mg	13.2	6.9	195	66	59%
				BOR+DEX	BOR: 1.3 mg/m^2<br>DEX: 20 mg	16.5	7.4	207	67	56%
Dimopoulos_ Haematologica_2018 Jonathan_ASH_2019 (abstract)	POLLUX (NCT02076009)	P3, RCT, open-label, multicenter	previously treated MM with at least 1 prior LOT	DAR+LEN+DEX	LEN: 25 mg; DEX: 40 mg; DAR: 16mg/kg	51.3	34.3	286	65	NR
				LEN+DEX	LEN: 25 mg; DEX: 40 mg	51.3	16.0	283	65	NR
Mateos_ Haematologica_2017	TOURMALINE-MM1 (NCT01564537)	P3, RCT, double blind, multicenter	previously treated MM with 1 to 3 prior LOT	IXA+LEN+DEX	IXA: 4 mg; LEN: 25mg; DEX: 40mg (LEN: 10mg if creatinine clearance <60mL/min)	14.8	NR	360	66	58%*
				LEN+DEX	PBO: 4 mg; LEN: 25mg; DEX: 40mg (LEN: 10mg if creatinine clearance <60mL/min)	14.6	NR	362	66	56%*
Spencer_ Haematologica_2018 Katja_ASH_2019 (abstract)	CASTOR (NCT02136134)	P3, RCT, open-label, multicenter	previously treated MM with at least 1 prior LOT	DAR+BOR+DEX	BOR: 1.3mg/m^2;DEX: 20mg; DAR: 16mg/kg	47.0	13.4	251	64	NR
				BOR+DEX	BOR: 1.3mg/m^2; DEX: 20mg	47.0	5.2	247	64	NR
Kropff_AH_2017	NCT00813150	P3, RCT, open-label, multicenter	previously treated MM with 1 to 3 prior LOT	BOR+DEX	BOR: 1.3mg/m^2; DEX: 20mg	24.00	3.6	46	68	54%
				CYC+BOR+DEX	BOR:1.3mg/m^2; DEX=20mg;CYC=50mg	24.00	3.6	47	71	55%
Jakubowiak_Blood_2016	NCT01478048	P2, RCT, open-label, multicenter	previously treated MM with 1 to 3 prior LOT	ELO+BOR+DEX	ELO: 10mg/kg; BOR: 1.3mg/m^2;DEX: 16mg and 20mg	15.9	NR	77	65	55%
				BOR+DEX	BOR: 1.3mg/m^2; DEX: 20mg	11.7	NR	75	65	49%
Richardson_LO_2019	OPTIMISMM (NCT01734928)	P2, RCT, open-label, multicenter	previously treated MM with 1 to 3 prior LOT	POM+BOR+DEX	POM: 4 mg; BOR: 1.3 mg/m^2DEX: 20 mg	15.9	8.8	281	67	55%
				BOR+DEX	BOR: 1.3 mg/m^2; DEX: 20 mg	15.9	4.9	278	68	53%
Moreau_Leukemia_2017 (Subgroup 1 prior LOT))	ENDEAVOR (NCT01568866)	P3, RCT, open-label, multicenter	previously treated MM with 1 to 3 prior LOT	CAR+DEX	CAR: 20mg/m^2 days 1-2 (cycle 1),56mg/m^2 thereafter; DEX: 20mg	NR	9.6	232	60	NR
				BOR+DEX	BOR: 1.3mg/m^2; DEX: 20mg	NR	7.1	232	64	NR

* Numbers are from the same trial but old references, as the updated reference did not report those baseline values.
** Weber (207) and Dimopoulos (2007) were both majority 3L+ populations, but were required in the 2L network to link LEN+DEX with DEX.

Table S1. Study Characteristics (2L Population)

Short Reference	Study Acronym	Study Design	Study Population	Interventions Acronym	Tx Dosage Included (mg)	Follow-Up (median months)	Duration of Treatment (median months)	N	Age (median)	Male (%)
Iida_CS_2018	JCOG0904, UMIN000003135	P2, RCT, open-label, multicenter	Japanese adults (≥20<80) with MM who received at least 1 prior LOT	BOR+DEX	BOR: 1.3mg/m^2; DEX: 20mg	34.3	NR	22	65	45%
				THAL+DEX	THAL: 200mg/d; DEX: 20mg	34.3	NR	22	67	59%
Yong_EHA_2018 (poster)	MUK Five	P2, RCT, open-label, multicenter	previously treated MM with 1 prior LOT	CAR+CYC+DEX	CAR: 20 & 36 mg/m^2; CYC: 500mg; DEX: 40 mg	14.0	5.5	141	NR	NR
				BOR+CYC+DEX	BOR: 1.3 mg/m^2; CYC: 500mg; DEX: 40 mg	14.0	5.5	99	NR	NR
Montefusco_BJH_2020	EUDRACT 2010-021557-40	P3, RCT, open-label, multicenter	MM at first relapse	LEN+CYC+DEX	LEN: 15 mg; CYC:500 mg/m^2; DEX: 20 mg	32.0	8.3*	79	64	55.7%
				BOR+CYC+DEX	BOR: 1.3mg/m^2; CYC: 500 mg/m^2; DEX: 20 mg	34.0	10.4*	76	65	36.8%
White_Cancer_2013	AMBER (NCT00473590)	P2, RCT, open label, multi-center	1-3 prior therapies	BEV+BOR	BOR: 1.3 mg/m2; BEV: 15 mg/kg	13.3	4.2	49	65	59%
				BOR	BOR: 1.3 mg/m2	13.3	NR	53	65	57%
Richardson_NEJM_2005	APEX	P3, RCT, open label, multi-center	1-3 prior therapies	BOR	BOR: 1.3 mg/m^2	8.0	NR	333	62	56%
				DEX	DEX: 40 mg	8.0	NR	336	61	60%
Weber_NEJM_2007**	MM-009 (NCT00056160)	P3, RCT, double blind, multicenter	≥1 prior therapy	LEN+DEX	LEN: 25 mg; DEX: 40mg	26.2	10.2	177	64	60%
				PBO+DEX	DEX: 40mg	12.9	NR	176	62	59%
Dimopoulos_NEJM_2007**	MM-010 (NCT00424047)	P3, RCT, double blind, multicenter	≥1 prior therapy	LEN+DEX	LEN: 25 mg; DEX: 40mg	16.4	10.2	176	63	59%
				PBO+DEX	DEX: 40mg	16.4	NR	175	64	58%
Orlowski_AJH_2015	NR	P2, RCT, double blind, multicenter	1-3 prior therapies	SIL+BOR	SIL: 6 mg/kg; BOR: 1.3 mg/m2	24.5	4.7	142	64	51%
				BOR	BOR: 1.3 mg/m2	24.5	4.7	144	61	59%
Dimopoulos_LO_2013	VANTAGE 088 (NCT00773747)	P3, RCT, double blind, multicenter	1-3 prior therapies	VOR+BOR	BOR: 1.3 mg/m²; VOR: 400 mg	14.2	NR	317	61	60%
				BOR	BOR: 1.3 mg/m²	NR	NR	320	63	58%
Siegel_JCO_2018	ASPIRE (NCT01080391)	P3, RCT, open-label, multicenter	previously treated MM with 1 to 3 prior LOT	CAR+LEN+DEX	CAR: 20 & 27 mg/m^2; LEN: 25 mg; DEX: 40 mg	67.1	20.1*	396	63*	NR
				LEN+DEX	LEN: 25 mg; DEX: 40 mg	67.1	13.1*	396	65*	NR

* Numbers are from the same trial but old references, as the updated reference did not report those baseline values.
** Weber (207) and Dimopoulos (2007) were both majority 3L+ populations, but were required in the 2L network to link LEN+DEX with DEX.

Table S1. Study Characteristics (2L Population)

Short Reference	Study Acronym	Study Design	Study Population	Interventions Acronym	Tx Dosage Included (mg)	Follow-Up (median months)	Duration of Treatment (median months)	N	Age (median)	Male (%)
San-Miguel_Lancet_2016	PANORAMA 1 (NCT01023308)	P3, RCT, double blind, multicenter	previously treated MM with 1 to 3 prior LOT	PAN+BOR+DEX	PAN: 20 mg; BOR: 1.3 mg/m^2; DEX: 20 mg	NR	NR	387	63	52%
				Vd	BOR: 1.3 mg/m^2; DEX: 20 mg	NR	NR	381	63	54%
Orlowski_CLML_2019	ENDEAVOR (NCT01568866)	P3, RCT, open-label, multicenter	previously treated MM with 1 to 3 prior LOT	CAR+DEX	CAR: 20mg/m^2 days 1-2 (cycle 1), 56mg/m^2 there-after; DEX: 20mg	44.3	11.1	464	65	NR
				Vd	BOR: 1.3mg/m^2; DEX: 20mg	43.7	6.2	465	65	NR
Chiou_CI_2007	NR	P2, RCT, open label, multi-center,	1 prior therapies	THAL+InfA	THAL: 200 mg/day up to 800 mg/day; INF: 3 MIU/m2 subcutaneous injection thrice weekly,	NR	NR	16	64	81%
				THAL	THAL: 200 mg/day up to 800 mg/day	NR	NR	12	62	92%
Richardson_Blood_2016 (≥2 prior LOT including BTZ and an IMiD)	PANORAMA 1 (NCT01023308)	P3, RCT, double blind, multicenter	previously treated MM with 1 to 3 prior LOT	PAN+BOR+DEX	PAN 20 mg + BOR 1.3 mg/m2 + DEX 20 mg	NR	6.4	73	61	56%
				Vd	PBO + BOR 1.3 mg/m2 + DEX 20 mg	NR	4.9	74	61	45%
Moreau_ASH_2019 (abstract)	BELLINI (NCT02755597)	P3, RCT, double blind, multicenter	previously treated MM with 1 to 3 prior LOT	VEN+BOR+DEX	VEN: 800 mg; BOR: 1.3 mg/m^2; DEX: 20 mg	22.7	NR	194	66	NR
				PBO+BOR+DEX	BOR: 1.3 mg/m^2; DEX: 20 mg	22.7	NR	97	66	NR
Hus_AH_2011	NR	RCT, multi-center	≥1 prior therapy	LOV+THAL+DEX	LOV: 2mg/kg / 0.5 mg/kg ; THAL: 100 mg ; DEX: 20 mg	NR	NR	49	59	49%
				THAL+DEX	THAL: 100 mg; DEX: 20 mg	NR	NR	42	62	48%
Dimopoulos_Haematolog-ica_2015	patients with one prior line in MMY-2045 (NCT00908232), APEX (NCT00048230), and DOX-IL-MMY-3001 (NCT00103506)	Retrospective matched-pairs analysis	1 prior therapy	BOR+DEX	median cumulative doses of bortezomib was 27.02 mg/m2 (range 1.3- 41.7 mg/m2); median cumulative dose of dexamethasone of 880 mg (range 20-1280 mg)	26.1	4.5	109	62	NR
				BOR	median cumulative doses of bortezomib was 28.60 mg/m2 (range 2.4-59.1 mg/m2)	18.4	4.1	109	64	NR

* Numbers are from the same trial but old references, as the updated reference did not report those baseline values.
** Weber (207) and Dimopoulos (2007) were both majority 3L+ populations, but were required in the 2L network to link LEN+DEX with DEX.

Table S1. Study Characteristics (2L Population)

Short Reference	Study Acronym	Study Design	Study Population	Interventions Acronym	Tx Dosage Included (mg)	Follow-Up (median months)	Duration of Treatment (median months)	N	Age (median)	Male (%)
Dimopoulos_Cancer_2018	ELOQUENT-2 (NCT01239797)	P3, RCT, open-label, multicenter	previously treated MM with 1 to 3 prior LOT	ELO+LEN+DEX	NR	46	NR	319	67	60%*
				LEN+DEX	NR		NR	316		59%*
Usmani_Blood_2019 (abstract)<br><br>Weisel_EHA_2020 (abstract)	CANDOR (NCT03158688)	P3, RCT, open-label, multicenter	previously treated MM with 1-3 prior LOT	DAR+CAR+DEX	DAR: 8 mg/kg then 16 mg/kg<br>CAR: 20 mg/m2 on cycle 1 days 1 and 2 and 56 mg/m2 beginning on cycle 1 day 8 and thereafter<br>DEX: 40 mg	17	16.14144737	312	64	NR
				CAR+DEX	CAR: 20 mg/m^2 on cycle 1 days 1 and 2 and 56 mg/m^2 beginning on cycle 1 day 8 and thereafter<br>DEX: 40 mg		9.279605263	154		

* Numbers are from the same trial but old references, as the updated reference did not report those baseline values.
** Weber (207) and Dimopoulos (2007) were both majority 3L+ populations, but were required in the 2L network to link LEN+DEX with DEX.

Table S2. Study Characteristics (3L+ Population)

Short Reference	Study Acronym	Study Design	Study Population	Interventions Acronym	Tx Dosage Included (mg)	Follow-Up (median months)	Duration of Treatment (median months)	N	Age (median)	Male (%)
Dimopoulos_ASCO_2020 (abstract)	BOSTON (NCT03110562)	P3, RCT, open-label, multicenter	previously treated MM with 1 to 3 prior LOT	SEL+BOR+DEX	SEL: 100 mg BOR: 1.3 mg/m^2 DEX: 20 mg	13.2	6.9	195	66	59%
				BOR+DEX	BOR: 1.3 mg/m^2 DEX: 20 mg	16.5	7.4	207	67	56%
Dimopoulos_Cancer_2018	ELOQUENT-2 (NCT01239797)	P3, RCT, open-label, multicenter	previously treated MM with 1 to 3 prior LOT	ELO+LEN+DEX	NR	46	NR	319	67	NR
				LEN+DEX	NR	46	NR	316	67	NR
Hou_JHO_2017	TOURMALINE-MM1 (NCT01564537)	P3, RCT, double blind, multicenter	previously treated MM with 1 to 3 prior LOT	IXA+LEN+DEX	IXA: 4 mg; LEN: 25mg; DEX: 40mg (LEN: 10mg if creatinine clearance <60mL/min)	20	9.1	57	61	41%
				LEN+DEX	PBO: 4 mg; LEN: 25mg; DEX: 40mg (LEN: 10mg if creatinine clearance <60mL/min)	20	6	58	62	38%
Bernal-Mizrachi_ASH_2019 (abstract) (NFKB2 FISH [+] subgroup)	NCT02765854	P2, RCT, open-label, multicenter	Patients with early relapsed multiple myeloma with NFKB2 FISH [+], who received <4 prior LOT	LEN+IXA+DEX	LEN: 25 mg; IXA: 4 mg; DEX: 40 mg	NR	NR	12	NR	NR
				IXA+DEX	IXA: 4 mg; DEX: 40 mg	NR	NR	13	NR	NR
Raje_BJH_2017	NCT01602224	P2, RCT, double blind, multicenter	previously treated MM with 1 to 3 prior LOT	TAB+BOR+DEX	TAB: 100 mg; BOR: 1.3 mg/m2; DEX: 20 mg	NR	NR	74	64	30%
				TAB+BOR+DEX	TAB: 300 mg; BOR: 1.3 mg/m^2; DEX: 20 mg	NR	NR	74	67	37%
				BOR+DEX	BOR: 1.3 mg/m^2; DEX: 20 mg	NR	NR	72	67	40%
Moreau_Leukemia_2017 (Subgroup ≥2 prior LOT)	ENDEAVOR (NCT01568866)	P3, RCT, open-label, multicenter	previously treated MM with 1 to 3 prior LOT	CAR+DEX	CAR: 20mg/m^2 days 1-2 (cycle 1), 56mg/m^2 thereafter; DEX: 20mg	NR	8.8	232	64	NR
				BOR+DEX	BOR: 1.3mg/m^2; DEX: 20mg	NR	6.7	233	66	NR

* Numbers are from the same trial but old references, as the updated reference did not report those baseline values.

Table S2. Study Characteristics (3L+ Population)

Short Reference	Study Acronym	Study Design	Study Population	Interventions Acronym	Tx Dosage Included (mg)	Follow-Up (median months)	Duration of Treatment (median months)	N	Age (median)	Male (%)
Yong_Blood_2017 (slides)	MUK Five	P2, RCT, open-label, multicenter	previously treated MM with 1 prior LOT	CAR+CYC+DEX	CAR: 20 & 36 mg/m^2; CYC: 500mg; DEX: 40 mg	NR	NR	201	67	58%
				BOR+CYC+DEX	BOR: 1.3 mg/m^2; CYC: 500mg; DEX: 40 mg	NR	NR	99	69	65%
Attal_Lancet_2019	ICARIA-MM (NCT02990338)	P3, RCT, open-label, multicenter	previously treated MM with at least 2 prior LOT	ISA+POM+DEX	ISA: 10mg/kg; POM: 4mg; DEX: 40mg	11.6	9.4	154	68	58%
Richardson_ASCO_2019 (abstract)				POM+DEX	POM: 4mg; DEX: 40mg	11.6	5.5	153	66	46%
Bringhen_CLML_2019 (abstract) (Subgroup >3 prior LOT)	ICARIA-MM (NCT02990338)	P3, RCT, open-label, multicenter	previously treated MM with at least 2 prior LOT	ISA+POM+DEX	ISA: 10mg/kg; POM: 4mg; DEX: 40mg	11.6	NR	52	NR	NR
				POM+DEX	POM: 4mg; DEX: 40mg	11.6	NR	52	NR	NR
Mateos_LH_2019	KEYNOTE-183 (NCT02576977)	P3, RCT, open-label, multicenter	previously treated MM with at least 2 prior LOT	PEM+POM+DEX	PEM: 200 mg; POM: 4 mg; DEX: 40 mg	NR	4.1	126	66	62%
				POM+DEX	POM: 4 mg; DEX: 40mg	NR	4.2	125	64	63%
Dimopoulos_NEJM_2018	ELOQUENT-3 (NCT02654132)	P2, RCT, open-label, multicenter	previously treated MM with at least 2 prior LOT	ELO+POM+DEX	ELO: 10 mg/kg; POM: 4 mg; DEX: 40 mg (20 mg if >75 years)	NR	NR	60	69	53%
Dimopoulos_CLML_2019 (abstract)				POM+DEX	POM: 4 mg; DEX: 40 mg (20 mg if >75 years)	NR	NR	57	66	61%
NCT01794039	NCT01794039	P2, RCT, open-label, single center	Patients ≥ 18 years old with relapsed myeloma that previously became refractory to lenalidomide	LEN+DEX	NR	NR	NR	5	67	60%
				POM+DEX	NR	NR	NR	4	69	75%
Richardson_Blood_2007	APEX	P3, RCT, open label, multicenter	1-3 prior therapies	BOR	BOR: 1.3 mg/m^2	22	4.1	333	62	56%
				DEX	DEX: 40 mg	22	4.1	336	61	60%
Orlowski_JCO_2007	NCT00103506	P3, RCT, open label, multicenter	≥1 prior therapy	PLD+BOR	PLD: 30 mg/m2; BOR: 1.3 mg/m2	NR	3.5	322	61	58%
				BOR	BOR: 1.3 mg/m2	NR	3.5	324	62	54%
Richardson_Blood_2014	MM-002 (NCT00833833)	P2, RCT, open label, multicenter	≥1prior therapy	POM+LoDEX	POM: 4 mg/day ; DEX: 40 mg/week	14.2	NR	113	63	54%
				POM	POM: 4 mg/day	14.2	NR	108	63	54%

* Numbers are from the same trial but old references, as the updated reference did not report those baseline values.

Table S2. Study Characteristics (3L+ Population)

Short Reference	Study Acronym	Study Design	Study Population	Interventions Acronym	Tx Dosage Included (mg)	Follow-Up (median months)	Duration of Treatment (median months)	N	Age (median)	Male (%)
Dimopoulos_ Haematologica_2015	MM-003 (NCT01311687)	P3, RCT, open label, multicenter, subgroup analysis	≥2 prior therapies	POM+LoDEX	POM: 4 mg/day; DEX: 40 mg/day	15.4	3.5	302	64	NR
				HiDEX	DEX: 40 mg/day	15.4	NR	153	65	NR
Offidani_HJ_2004	NR	P2, RCT, open label, multicenter,	≥1 prior therapies	THAL+MEL	THAL: 100 mg/day - 600 mg/day; MEL: 0.20 mg/kg/day	NR	NR	23	69	37%
				THAL	THAL: 100 mg/day- up to 600 mg/day	NR	NR	23	74	43%
Hjorth_EJH_2012	NCT00602511	P3, RCT, open label, multicenter	≥1 prior therapies	THAL+DEX	THAL: 50 mg; DEX: 40 mg	NR	5.1	67	71	42%
				BOR+DEX	BOR: 1.3 mg/m; DEX: 20 mg	NR	3.5	64	71	64%
Dimopoulos_ Haematologica_2015	patients with one prior line in MMY-2045 (NCT00908232), APEX (NCT00048230), and DOXIL-MMY-3001 (NCT00103506)	Retrospective matched-pairs analysis	1 prior therapy	BOR+DEX	median cumulative doses of bortezomib was 27.02 mg/m2 (range 1.3- 41.7 mg/m2); median cumulative dose of dexamethasone of 880 mg (range 20-1280 mg)	26.1	4.5	109	62	NR
				BOR	median cumulative doses of bortezomib was 28.60 mg/m2 (range 2.4-59.1 mg/m2)	18.4	4.1	109	64	NR
Baz_Blood_2016	NCT01432600	P2, RCT, open-label, multicenter	previously treated MM with at least 2 prior LOT	POM+DEX	POM: 4 mg<br>DEX: 40 mg	NR	NR	36	64	0.64
				CYC+POM+DEX	CYC: 400 mg<br>POM: 4 mg<br>DEX: 40 mg		NR	34	65	0.53
Bringhen_CLML_2019 (abstract) (Subgroup 2-3 prior LOT)	ICARIA-MM (NCT02990338)	P3, RCT, open-label, multicenter	previously treated MM with at least 2 prior LOT	ISA+POM+DEX	ISA: 10mg/kg<br>POM: 4mg<br>DEX: 40mg	11.6	NR	102	NR	NR
				POM+DEX	POM: 4mg<br>DEX: 40mg		NR	101	NR	NR

* Numbers are from the same trial but old references, as the updated reference did not report those baseline values.

Table S2. Study Characteristics (3L+ Population)

Short Reference	Study Acronym	Study Design	Study Population	Interventions Acronym	Tx Dosage Included (mg)	Follow-Up (median months)	Duration of Treatment (median months)	N	Age (median)	Male (%)
Croft_ASH_2018 (abstract)	Muk seven (NCT02406222)	P2, RCT, open-label, multicenter	previously treated MM with at least 2 prior LOT	CYC+POM+DEX	CYC: 500 mg<br>POM: 4 mgDEX: 40 mg (20 mg if >75 years)	NR	NR	51	NR	NR
				POM+DEX	POM: 4 mg<br>DEX: 40 mg (20 mg if >75 years)		NR	51	NR	NR
Dimopoulos_ Haematologica_2018<br>Jonathan_ASH_2019 (abstract)	POLLUX (NCT02076009)	P3, RCT, open-label, multicenter	previously treated MM with at least 1 prior LOT	DAR+LEN+DEX	LEN: 25 mg<br>DEX: 40 mg<br>DAR: 16mg/kg	51.3	34.3	286	65	NR
				LEN+DEX	LEN: 25 mg<br>DEX: 40 mg		16	283	65	NR
Dimopoulos_LO_2013	VANTAGE 088 (NCT00773747)	P3, RCT, double blind, multicenter	1-3 prior therapies	VOR+BOR	BOR: 1.3 mg/m²<br>VOR: 400 mg	14.2	NR	317	60.9	60%
				PBO+BOR	BOR: 1.3 mg/m²	NR	NR	320	62.7	58%
Dimopoulos_NEJM_2007	MM-010 (NCT00424047)	P3, RCT, double blind, multicenter	≥1 prior therapy	LEN+DEX	LEN: 25 mg<br>DEX: 40mg	16.4	10.15384615	176	63	59%
				PBO+DEX	DEX: 40mg		NR	175	64	58%
Hajek_Leukemia_2017	FOCUS (NCT01302392)	P3, RCT, open-label, multicenter	previously treated MM with at least 3 prior LOT	CAR	CAR: 20 mg	27.8	16.3	157	63	52%
				COR+/-CYC	COR (Prednisone: 30 mg, DEX: 6 mg)<br>CYC: 50 mg	29.8	10.7	158	66	61%
Huang_EHA_2020 (abstract)	MMY3009 (LEPUS)	P3, RCT, multicenter	previously treated MM with at least 1 prior LOT	DAR+BOR+DEX	DAR: 16 mg/kg<br>BOR: 1.3 mg/m^2<br>DEX: 20 mg	8.2	NR	141	61	NR
				BOR+DEX	BOR: 1.3 mg/m^2<br>DEX: 20 mg		NR	70	61	NR
Jakubowiak_Blood_2016	NCT01478048	P2, RCT, open-label, multicenter	previously treated MM with 1 to 3 prior LOT	ELO+BOR+DEX	ELO: 10mg/kg<br>BOR: 1.3mg/m^2<br>DEX: 16mg and 20mg	15.9	NR	77	65	55%
				BOR+DEX	BOR: 1.3mg/m^2<br>DEX: 20mg	11.7	NR	75	65	49%
Kropff_Haematologica_2012	OPTIMUM (NCT00452569)	P3, RCT, open label, multicenter	1-3 prior therapies	THAL 100	THAL: 100 mg/day	NR	6.447368421	121	64	45%
				THAL 200	THAL: 200 mg/day	NR	6.447368421	122	63	46%
				THAL 400	THAL: 400 mg/day	NR	6.447368421	130	65	59%
				DEX	DEX: 40 mg/day	NR	5.526315789	126	63	45%

* Numbers are from the same trial but old references, as the updated reference did not report those baseline values.

Table S2. Study Characteristics (3L+ Population)

Short Reference	Study Acronym	Study Design	Study Population	Interventions Acronym	Tx Dosage Included (mg)	Follow-Up (median months)	Duration of Treatment (median months)	N	Age (median)	Male (%)
Kumar_EHA_2020 (abstract), Moreau_ASH_2019 (abstract)	BELLINI (NCT02755597)	P3, RCT, double blind, multicenter	previously treated MM with 1 to 3 prior LOT	VEN+BOR+DEX	VEN: 800 mg<br>BOR: 1.3 mg/m^2<br>DEX: 20 mg	28.6	NR	194	66	NR
				PBO+BOR+DEX	BOR: 1.3 mg/m^2<br>DEX: 20 mg		NR	97	66	NR
Mateos_Haematologica_2017	TOURMALINE-MM1 (NCT01564537)	P3, RCT, double blind, multicenter	previously treated MM with 1 to 3 prior LOT	IXA+LEN+DEX	IXA: 4 mg<br>LEN: 25mg<br>DEX: 40mg<br>(LEN: 10mg if creatinine clearance <60mL/min)	14.8	NR	360	NR	NR
				PBO+LEN+DEX	PBO: 4 mg<br>LEN: 25mg<br>DEX: 40mg<br>(LEN: 10mg if creatinine clearance <60mL/min)	14.6		362	NR	NR
Orlowski_CLML_2019	ENDEAVOR (NCT01568866)	P3, RCT, open-label, multicenter	previously treated MM with 1 to 3 prior LOT	CAR+DEX	CAR: 20mg/m^2 days 1-2 (cycle 1), 56mg/m^2 thereafter<br>DEX: 20mg	44.3	11.1	464	65	NR
				BOR+DEX	BOR: 1.3mg/m^2<br>DEX: 20mg	43.7	6.2	465	65	NR
Richardson_Blood_2016 (≥2 prior LOT including BTZ and an IMiD)	PANORAMA 1 (NCT01023308)	P3, RCT, double blind, multicenter	previously treated MM with 1 to 3 prior LOT	PAN+BOR+DEX	PAN 20 mg + BOR 1.3 mg/m2 + DEX 20 mg	NR	6.4	73	61	56%
				PBO+BOR+DEX	PBO + BOR 1.3 mg/m2 + DEX 20 mg	NR	4.9	74	61	45%
Richardson_LO_2019	OPTIMISMM (NCT01734928)	P3, RCT, open-label, multicenter	previously treated MM with 1 to 3 prior LOT	POM+BOR+DEX	POM: 4 mg<br>BOR: 1.3 mg/m^2DEX: 20 mg	15.9	8.8	281	67	55%
				BOR+DEX	BOR: 1.3 mg/m^2<br>DEX: 20 mg		4.9	278	68	53%
San-Miguel_Lancet_2016	PANORAMA 1 (NCT01023308)	P3, RCT, double blind, multicenter	previously treated MM with 1 to 3 prior LOT	PAN+BOR+DEX	PAN: 20 mg<br>BOR: 1.3 mg/m^2<br>DEX: 20 mg	NR	NR	387	63	52%
				PBO+BOR+DEX	BOR: 1.3 mg/m^2<br>DEX: 20 mg	NR	NR	381	63	54%

* Numbers are from the same trial but old references, as the updated reference did not report those baseline values.

Table S2. Study Characteristics (3L+ Population)

Short Reference	Study Acronym	Study Design	Study Population	Interventions Acronym	Tx Dosage Included (mg)	Follow-Up (median months)	Duration of Treatment (median months)	N	Age (median)	Male (%)
Siegel_JCO_2018	ASPIRE (NCT01080391)	P3, RCT, open-label, multicenter	previously treated MM with 1 to 3 prior LOT	CAR+LEN+DEX	CAR: 20 & 27 mg/m^2<br>LEN: 25 mg<br>DEX: 40 mg	67.1	20.1*	396	63*	NR
				LEN+DEX	LEN: 25 mg<br>DEX: 40 mg		13.1*	396	65*	NR
Spencer_Haematologica_2018<br>Katja_ASH_2019 (abstract)	CASTOR (NCT02136134)	P3, RCT, open-label, multicenter	previously treated MM with at least 1 prior LOT	DAR+BOR+DEX	BOR: 1.3mg/m^2<br>DEX: 20mg<br>DAR: 16mg/kg	47.0	13.4	251	64	NR
				BOR+DEX	BOR: 1.3mg/m^2<br>DEX: 20mg		5.2	247	64	
Usmani_Blood_2019 (abstract)<br>Weisel_EHA_2020 (abstract)	CANDOR (NCT03158688)	P3, RCT, open-label, multicenter	previously treated MM with 1-3 prior LOT	DAR+CAR+DEX	DAR: 8 mg/kg then 16 mg/kg<br>CAR: 20 mg/m2 on cycle 1 days 1 and 2 and 56 mg/m2 beginning on cycle 1 day 8 and thereafter<br>DEX: 40 mg	17	16.1	312	64	NR
				CAR+DEX	CAR: 20 mg/m^2 on cycle 1 days 1 and 2 and 56 mg/m^2 beginning on cycle 1 day 8 and thereafter<br>DEX: 40 mg		9.3	154		
Verkleij_Blood_2019 (abstract)	NCT03184194	P2, RCT, multicenter	previously treated MM with at least 2 prior LOT	NIV+DAR+low dose CYC	DAR: 16 mg/kg<br>NIV: 240 mg in cycles 1-6 and 480 mg thereafter<br>Low-dose CYC: 50 mg	8.6	NR	20	64	45%
				NIV+DAR	DAR: 16 mg/kg<br>NIV: 240 mg in cycles 1-6 and 480 mg thereafter		NR	20	66	65%
Weber_NEJM_2007	MM-009 (NCT00056160)	P3, RCT, double blind, multicenter	≥1 prior therapy	LEN+DEX	LEN: 25 mg<br>DEX: 40mg	26.2	10.15	177	64	60%
				PBO+DEX	DEX: 40mg	12.9	NR	176	62	59%

* Numbers are from the same trial but old references, as the updated reference did not report those baseline values.

Table S3. 2L PFS Ranking Probability Results

Probability of Rank (%)	Treatment																				
	Vd	XVd	BOR	Rd	KRd	ERd	DRd	IRd	RCyd	DVd	EVd	PVd	VCyd	VOR+ BOR	BEV+ BOR	SIL+ BOR	DKd	Kd	KCyd	DEX	Td
Rank 1	0.0	0.0	0.0	0.0	0.0	0.0	3.3	0.0	0.0	83.1	0.0	0.0	0.0	0.0	0.0	0.0	13.6	0.0	0.0	0.0	0.0
Rank 2	0.0	0.6	0.0	0.0	0.1	0.0	19.2	0.0	0.0	15.7	0.6	3.2	0.0	0.0	0.0	0.0	56.6	3.8	0.0	0.0	0.0
Rank 3	0.0	2.6	0.0	0.0	2.1	0.6	25.4	0.2	0.0	1.1	2.6	10.6	0.0	0.0	0.1	0.0	17.6	36.9	0.0	0.0	0.0
Rank 4	0.0	6.9	0.0	0.0	5.1	2.1	18.3	0.8	0.1	0.1	6.4	20.6	0.0	0.0	0.2	0.0	5.9	33.4	0.0	0.0	0.0
Rank 5	0.0	14.4	0.0	0.1	8.3	4.3	14.3	2.0	0.2	0.0	12.7	25.0	0.0	0.1	0.5	0.0	2.9	14.9	0.2	0.0	0.1
Rank 6	0.1	20.7	0.0	0.3	12.2	7.5	10.1	3.8	0.5	0.0	18.4	16.5	0.0	0.4	1.0	0.0	1.6	6.0	0.8	0.0	0.1
Rank 7	1.9	18.5	0.0	1.0	15.5	11.4	6.8	6.4	1.3	0.0	18.5	9.9	0.2	1.1	1.9	0.0	0.8	2.7	1.9	0.0	0.4
Rank 8	10.4	10.2	0.0	2.6	18.6	15.6	1.5	10.1	2.4	0.0	10.6	5.4	0.6	2.5	3.2	0.1	0.4	1.3	3.7	0.0	0.8
Rank 9	10.4	7.8	0.0	5.7	13.3	17.0	0.5	13.1	3.2	0.0	8.2	3.7	1.6	4.2	4.0	0.3	0.2	0.6	5.1	0.0	1.3
Rank 10	11.6	6.3	0.0	10.6	8.4	13.0	0.3	14.4	3.8	0.0	6.7	2.4	3.0	6.3	5.0	0.7	0.1	0.3	5.6	0.0	1.5
**Average rank**	**11.64**	**7.24**	**18.21**	**12.76**	**8.07**	**9.29**	**3.93**	**10.84**	**15.49**	**1.18**	**7.54**	**5.46**	**16.05**	**13.49**	**14.37**	**16.44**	**2.42**	**4.01**	**14.12**	**20.76**	**17.69**
**Probability of being in top 5 (%)**	**0.0**	**24.5**	**0.0**	**0.1**	**15.7**	**7.1**	**80.6**	**3.1**	**0.3**	**100.0**	**22.4**	**59.4**	**0.0**	**0.2**	**0.8**	**0.0**	**96.6**	**89.0**	**0.3**	**0.0**	**0.1**

Abbreviations: Vd, bortezomib + dexamethasone; XVd, selinexor + bortezomib + dexamethasone; BOR, bortezomib; Rd, lenalidomide + dexamethasone; KRd, carfilzomib + lenalidomide + dexamethasone; ERd, elotuzumab + lenalidomide + dexamethasone; DRd, daratumumab + lenalidomide + dexamethasone; IRd, ixazomib + lenalidomide + dexamethasone; RCyd, lenalidomide + cyclophosphamide + dexamethasone; DVd, daratumumab + bortezomib + dexamethasone; EVd, elotuzumab + bortezomib + dexamethasone; PVd, pomalidomide + bortezomib + dexamethasone; VCyd, bortezomib + cyclophosphamide + dexamethasone; VOR+BOR, vorinostat + bortezomib; BEV+BOR, bevacizumab + bortezomib; SIL+BOR, siltuximab + bortezomib; DKd, daratumuab + carfilzomib + dexamethasone; Kd, carfilzomib + dexamethasone; KCyd, carfilzomib + cyclophosphamide + dexamethasone; DEX, dexamethasone; Td, thalidomide + dexamethasone.

Table S4. 2L OS Ranking Probability Results

Probability of Rank (%)	Treatment														
	Vd	XVd	BOR	Rd	KRd	ERd	IRd	EVd	VCyd	BEV+ BOR	SIL+ BOR	Kd	DEX	Td	FVd
Rank 1	0.0	11.4	0.1	0.0	0.9	0.4	26.6	28.1	1.4	26.6	0.0	3.2	0.0	0.7	0.5
Rank 2	0.0	17.7	1.0	0.1	4.5	1.9	15.3	23.4	2.9	17.7	0.1	11.4	0.0	1.4	2.6
Rank 3	0.3	16.8	2.8	0.7	7.1	3.6	9.7	15.3	4.0	11.0	0.4	19.3	0.0	1.8	7.2
Rank 4	2.4	13.1	5.2	2.3	7.3	4.9	6.9	9.3	4.9	7.8	0.7	20.3	0.0	2.2	12.8
Rank 5	8.6	9.6	7.3	3.6	6.9	5.2	5.6	6.4	5.5	6.2	1.3	15.2	0.0	2.5	16.0
Rank 6	15.8	7.5	9.6	4.2	6.8	5.3	4.9	4.8	6.0	5.4	2.0	10.0	0.1	2.8	14.8
Rank 7	17.6	6.1	12.7	4.5	6.9	5.5	4.9	3.6	7.2	5.2	2.9	7.1	0.1	3.8	12.0
Rank 8	14.3	4.8	16.5	5.1	8.1	6.2	5.3	2.7	7.9	4.7	4.8	5.2	0.2	4.7	9.5
Rank 9	11.2	3.8	16.4	6.0	10.2	7.7	5.1	2.0	8.2	3.7	8.4	3.7	0.3	5.5	7.7
Rank 10	9.9	2.9	11.8	8.6	12.3	10.2	4.6	1.5	7.8	2.9	11.9	2.4	0.5	6.2	6.4
**Average rank**	**8.18**	**4.63**	**8.06**	**10.80**	**8.05**	**9.59**	**4.77**	**3.33**	**9.41**	**4.35**	**11.35**	**4.75**	**14.50**	**11.58**	**6.65**
**Probability of being in top 5 (%)**	**11.4**	**68.6**	**16.3**	**6.7**	**26.8**	**16.0**	**64.1**	**82.5**	**18.7**	**69.3**	**2.6**	**69.4**	**0.0**	**8.6**	**39.1**

Abbreviations: Vd, bortezomib + dexamethasone; XVd, selinexor + bortezomib + dexamethasone; BOR, bortezomib; Rd, lenalidomide + dexamethasone; KRd, carfilzomib + lenalidomide + dexamethasone; ERd, elotuzumab + lenalidomide + dexamethasone; IRd, ixazomib + lenalidomide + dexamethasone; EVd, elotuzumab + bortezomib + dexamethasone; VCyd, bortezomib + cyclophosphamide + dexamethasone; BEV+BOR, bevacizumab + bortezomib; SIL+BOR, siltuximab + bortezomib; Kd, carfilzomib + dexamethasone; DEX, dexamethasone; Td, thalidomide + dexamethasone; FVd, panobinostat + bortezomib + dexamethasone.

Table S5. 2L ORR Ranking Probability Results

Probability of Rank (%)	Treatment														
	Vd	XVd	BOR	Rd	KRd	DRd	IRd	RCyd	DVd	EVd	VCyd	BEV+ BOR	SIL+ BOR	DKd	Kd
Rank 1	0.0	0.7	0.0	0.0	0.6	2.9	0.0	1.7	13.7	0.0	0.0	0.0	0.0	77.5	0.0
Rank 2	0.0	6.0	0.0	0.0	3.0	8.1	0.0	5.8	42.7	0.1	0.0	0.0	0.0	15.0	4.8
Rank 3	0.0	12.7	0.0	0.0	5.6	9.7	0.0	8.5	17.5	0.4	0.0	0.0	0.0	4.3	16.5
Rank 4	0.0	14.5	0.0	0.0	6.9	8.6	0.1	8.6	11.0	1.0	0.1	0.0	0.0	1.9	21.1
Rank 5	0.0	16.3	0.0	0.0	7.7	8.9	0.2	8.9	6.5	2.3	0.5	0.0	0.0	0.8	20.9
Rank 6	0.2	15.4	0.0	0.1	9.5	10.0	0.3	10.1	4.1	5.1	0.9	0.0	0.0	0.3	16.6
Rank 7	1.2	13.9	0.0	0.2	11.7	10.8	0.7	11.6	2.4	8.2	1.9	0.0	0.0	0.2	11.1
Rank 8	4.8	9.9	0.0	0.5	12.8	11.2	1.8	11.3	1.3	13.7	4.9	0.1	0.0	0.1	5.7
Rank 9	12.9	6.3	0.0	1.3	13.1	9.6	3.0	10.9	0.6	15.5	7.1	0.1	0.0	0.0	2.5
Rank 10	23.4	2.8	0.0	2.5	9.8	7.1	5.3	7.6	0.2	17.6	11.3	0.3	0.1	0.0	0.6
**Average rank**	**10.72**	**5.62**	**17.78**	**13.80**	**7.74**	**6.63**	**13.15**	**7.07**	**2.88**	**9.66**	**11.98**	**16.16**	**15.97**	**1.36**	**5.01**
**Probability of being in top 5 (%)**	**0.0**	**50.1**	**0.0**	**0.0**	**23.7**	**38.2**	**0.2**	**33.4**	**91.3**	**3.8**	**0.6**	**0.0**	**0.0**	**99.4**	**63.4**

Abbreviations: Vd, bortezomib + dexamethasone; XVd, selinexor + bortezomib + dexamethasone; BOR, bortezomib; Rd, lenalidomide + dexamethasone; KRd, carfilzomib + lenalidomide + dexamethasone; DRd, daratumumab + lenalidomide + dexamethasone; IRd, ixazomib + lenalidomide + dexamethasone; RCyd, lenalidomide + cyclophosphamide + dexamethasone; DVd, daratumumab + bortezomib + dexamethasone; EVd, elotuzumab + bortezomib + dexamethasone; VCyd, bortezomib + cyclophosphamide + dexamethasone; BEV+BOR, bevacizumab + bortezomib; SIL+BOR, siltuximab + bortezomib; DKd, daratumuab + carfilzomib + dexamethasone; Kd, carfilzomib + dexamethasone.

Table S6. 3L+ PFS Ranking Probability Results

Probability of Rank (%)	Treatment														
	Vd	XVd	BOR	Rd	KRd	ERd	DRd	IRd	DVd	EVd	PVd	VOR+ BOR	DKd	Kd	DEX
Rank 1	0.0	0.1	0.0	0.0	0.0	0.0	33.5	1.2	3.5	1.3	0.1	0.0	48.3	0.0	0.0
Rank 2	0.0	0.4	0.0	0.0	0.4	0.8	22.0	6.7	12.2	3.1	0.9	0.0	24.0	1.2	0.0
Rank 3	0.0	1.2	0.0	0.0	1.4	2.5	12.5	9.1	17.8	4.3	3.0	0.0	11.6	4.3	0.0
Rank 4	0.0	2.3	0.0	0.0	2.6	4.3	8.1	8.9	16.7	4.9	6.2	0.0	6.0	8.7	0.0
Rank 5	0.0	3.5	0.0	0.0	3.9	5.4	5.6	8.2	13.0	5.2	9.4	0.0	3.6	12.2	0.0
Rank 6	0.0	5.0	0.0	0.1	4.7	6.0	4.5	7.4	9.7	5.6	11.8	0.0	2.3	13.9	0.0
Rank 7	0.0	7.1	0.0	0.3	5.4	6.3	4.1	7.2	7.6	6.2	13.0	0.0	1.6	13.3	0.0
Rank 8	0.2	9.8	0.0	0.7	5.9	6.9	3.9	7.7	5.9	7.4	12.2	0.0	1.0	11.3	0.0
Rank 9	1.4	10.7	0.0	1.1	7.0	8.0	3.1	8.8	4.5	7.7	10.2	0.0	0.7	9.1	0.0
Rank 10	4.2	9.3	0.0	1.4	8.6	9.9	1.5	9.4	3.1	6.8	8.4	0.0	0.4	7.4	0.0
**Average rank**	**15.23**	**10.74**	**20.87**	**15.97**	**11.03**	**10.09**	**3.20**	**7.71**	**5.17**	**10.38**	**8.32**	**19.82**	**2.24**	**7.66**	**23.95**
**Probability of being in top 5 (%)**	**0.0**	**7.6**	**0.0**	**0.0**	**8.2**	**13.2**	**81.6**	**34.1**	**63.2**	**18.7**	**19.6**	**0.0**	**93.4**	**26.4**	**0.0**

Abbreviations: Vd, bortezomib + dexamethasone; XVd, selinexor + bortezomib + dexamethasone; BOR, bortezomib; Rd, lenalidomide + dexamethasone; KRd, carfilzomib + lenalidomide + dexamethasone; ERd, elotuzumab + lenalidomide + dexamethasone; DRd, daratumumab + lenalidomide + dexamethasone; IRd, ixazomib + lenalidomide + dexamethasone; DVd, daratumumab + bortezomib + dexamethasone; EVd, elotuzumab + bortezomib + dexamethasone; PVd, pomalidomide + bortezomib + dexamethasone; VOR+BOR, vorinostat + bortezomib; BEV+BOR, bevacizumab + bortezomib; DKd, daratumuab + carfilzomib + dexamethasone; Kd, carfilzomib + dexamethasone; DEX, dexamethasone.

Table S7. 3L+ OS Ranking Probability Results

	Treatment														
**Probability of Rank (%)**	**Vd**	**XVd**	**BOR**	**Rd**	**KRd**	**ERd**	**IRd**	**DVd**	**PVd**	**VOR+BOR**	**DKd**	**Kd**	**DEX**	**FVd**	**ISA+Pd**
Rank 1	0.0	0.1	0.0	0.0	0.1	1.3	39.1	52.0	0.0	0.0	4.3	0.0	0.0	0.0	0.2
Rank 2	0.0	0.9	0.0	0.0	1.5	10.2	33.7	24.7	0.1	0.3	18.8	0.5	0.0	0.1	1.4
Rank 3	0.0	2.5	0.0	0.0	6.8	21.3	13.7	9.3	0.4	1.3	22.9	5.5	0.0	0.4	4.0
Rank 4	0.1	4.1	0.0	0.6	14.1	19.7	5.6	4.8	1.3	2.9	13.8	12.4	0.0	1.4	6.7
Rank 5	0.5	5.1	0.2	3.3	16.3	14.1	2.9	3.0	2.9	4.7	10.1	12.6	0.0	3.2	8.8
Rank 6	1.7	5.6	0.5	7.2	14.1	9.3	1.6	2.0	4.3	6.5	7.9	11.9	0.0	4.9	9.9
Rank 7	3.9	5.8	1.3	10.4	10.6	6.2	1.0	1.4	5.4	8.0	6.1	10.9	0.0	6.0	9.5
Rank 8	5.8	6.1	2.7	10.6	7.6	4.3	0.7	0.8	6.4	9.1	4.7	9.9	0.0	7.0	8.5
Rank 9	7.1	6.2	4.7	9.5	5.7	3.3	0.6	0.6	7.2	9.2	3.0	8.7	0.0	7.6	7.0
Rank 10	8.1	6.0	6.9	8.1	4.9	3.0	0.4	0.4	7.5	8.9	2.2	6.8	0.0	7.9	6.1
**Average rank**	**13.02**	**11.98**	**13.64**	**11.05**	**7.20**	**5.22**	**2.25**	**2.13**	**12.53**	**10.67**	**4.75**	**7.74**	**19.99**	**12.10**	**9.47**
**Probability of being in top 5 (%)**	**0.5**	**12.8**	**0.2**	**3.9**	**38.8**	**66.4**	**95.0**	**93.9**	**4.7**	**9.2**	**69.9**	**31.1**	**0.0**	**5.1**	**21.1**

Abbreviations: Vd, bortezomib + dexamethasone; XVd, selinexor + bortezomib + dexamethasone; BOR, bortezomib; Rd, lenalidomide + dexamethasone; KRd, carfilzomib + lenalidomide + dexamethasone; ERd, elotuzumab + lenalidomide + dexamethasone; IRd, ixazomib + lenalidomide + dexamethasone; DVd, daratumumab + bortezomib + dexamethasone; PVd, pomalidomide + bortezomib + dexamethasone; VOR+BOR, vorinostat + bortezomib; DKd, daratumuab + carfilzomib + dexamethasone; Kd, carfilzomib + dexamethasone; DEX, dexamethasone; FVd, panobinostat + bortezomib + dexamethasone; ISA+Pd, isatuximab + pomalidomide + dexamethasone.

Table S8. 3L+ ORR Ranking Probability Results

	Treatment														
**Probability of Rank (%)**	**Vd**	**XVd**	**BOR**	**Rd**	**KRd**	**ERd**	**DRd**	**IRd**	**DVd**	**PVd**	**VOR+BOR**	**DKd**	**Kd**	**DEX**	**FVd**
Rank 1	0.0	0.2	0.0	0.0	0.3	0.0	18.9	0.0	5.5	67.1	0.0	4.0	0.0	0.0	2.1
Rank 2	0.0	2.2	0.0	0.0	1.7	0.0	16.1	0.0	20.6	25.1	0.0	14.0	0.1	0.0	9.8
Rank 3	0.0	5.3	0.0	0.0	4.1	0.0	12.0	0.0	21.8	6.0	0.0	17.3	1.0	0.0	14.6
Rank 4	0.0	8.5	0.0	0.0	5.4	0.1	9.6	0.2	17.5	1.4	0.0	16.4	3.9	0.0	15.9
Rank 5	0.0	11.7	0.0	0.0	6.5	0.2	8.1	0.4	12.7	0.3	0.0	14.2	9.5	0.0	14.9
Rank 6	0.0	13.9	0.0	0.0	7.7	0.3	7.3	0.6	8.7	0.1	0.0	11.4	16.9	0.0	12.6
Rank 7	0.1	15.7	0.0	0.0	9.0	0.6	6.9	1.0	5.7	0.0	0.0	8.5	21.9	0.0	10.5
Rank 8	2.8	15.1	0.0	0.0	11.6	1.1	8.1	1.6	3.6	0.0	0.0	5.8	20.0	0.0	8.0
Rank 9	10.8	11.2	0.0	0.0	16.4	2.0	5.0	2.7	2.0	0.0	0.0	3.6	12.8	0.0	5.2
Rank 10	19.7	6.7	0.0	0.0	13.1	4.3	3.6	5.1	1.0	0.0	0.0	2.0	6.8	0.0	2.9
**Average rank**	**13.02**	**11.98**	**13.64**	**11.05**	**7.20**	**5.22**	**2.25**	**2.13**	**12.53**	**10.67**	**4.75**	**7.74**	**19.99**	**12.10**	**9.47**
**Probability of being in top 5 (%)**	**0.5**	**12.8**	**0.2**	**3.9**	**38.8**	**66.4**	**95.0**	**93.9**	**4.7**	**9.2**	**69.9**	**31.1**	**0.0**	**5.1**	**21.1**

Abbreviations: Vd, bortezomib + dexamethasone; XVd, selinexor + bortezomib + dexamethasone; BOR, bortezomib; Rd, lenalidomide + dexamethasone; KRd, carfilzomib + lenalidomide + dexamethasone; ERd, elotuzumab + lenalidomide + dexamethasone; DRd, daratumumab + lenalidomide + dexamethasone; IRd, ixazomib + lenalidomide + dexamethasone; DVd, daratumumab + bortezomib + dexamethasone; PVd, pomalidomide + bortezomib + dexamethasone; VOR+BOR, vorinostat + bortezomib; DKd, daratumuab + carfilzomib + dexamethasone; Kd, carfilzomib + dexamethasone; DEX, dexamethasone; FVd, panobinostat + bortezomib + dexamethasone.
